# Rapid gains in segmenting fluent speech when words match the rhythmic unit: evidence from infants acquiring syllable-timed languages

**DOI:** 10.3389/fpsyg.2013.00106

**Published:** 2013-03-05

**Authors:** Laura Bosch, Melània Figueras, Maria Teixidó, Marta Ramon-Casas

**Affiliations:** ^1^Department of Basic Psychology, University of BarcelonaBarcelona, Spain; ^2^Institute for Research in Brain, Behavior and Cognition (IR3C), University of BarcelonaBarcelona, Spain

**Keywords:** word segmentation, syllable-timed languages, natural speech, rhythmic unit, preference pattern, infants

## Abstract

The ability to extract word-forms from sentential contexts represents an initial step in infants' process toward lexical acquisition. By age 6 months the ability is just emerging and evidence of it is restricted to certain testing conditions. Most research has been developed with infants acquiring stress-timed languages (English, but also German and Dutch) whose rhythmic unit is not the syllable. Data from infants acquiring syllable-timed languages are still scarce and limited to French (European and Canadian), partially revealing some discrepancies with English regarding the age at which word segmentation ability emerges. Research reported here aims at broadening this cross-linguistic perspective by presenting first data on the early ability to segment monosyllabic word-forms by infants acquiring Spanish and Catalan. Three different language groups (two monolingual and one bilingual) and two different age groups (8- and 6-month-old infants) were tested using natural language and a modified version of the HPP with familiarization to passages and testing on words. Results revealed positive evidence of word segmentation in all groups at both ages, but critically, the pattern of preference differed by age. A novelty preference was obtained in the older groups, while the expected familiarity preference was only found at the younger age tested, suggesting more advanced segmentation ability with an increase in age. These results offer first evidence of an early ability for monosyllabic word segmentation in infants acquiring syllable-timed languages such as Spanish or Catalan, not previously described in the literature. Data show no impact of bilingual exposure in the emergence of this ability and results suggest rapid gains in early segmentation for words that match the rhythm unit of the native language.

## Introduction

The identification of possible word-forms within sentential contexts represents an initial step in infants' process toward lexical acquisition. Extracting word units from the input and detecting repetitions of these units in different contexts is considered a basic skill related to early vocabulary construction. Research has already shown an associative link between these early skills and later language outcomes (Newman et al., 2006; Junge et al., 2012; Singh et al., 2012). Characterizing the emergence of word segmentation ability is, thus, important in relation to the early building and growing of lexical knowledge. More specifically, exploring this emergent capacity in infants exposed to languages with different rhythmic structure offers the opportunity to identify differential features in the segmentation strategies used by infants, as well as possible variation in its developmental time-course. Finding evidence of variation in the time course for word segmentation might ultimately be useful to account for possible differences in early lexical acquisition processes from a cross-linguistic perspective. The present research addresses this issue by exploring early word segmentation abilities in infants exposed to Spanish and Catalan, two Romance languages whose rhythmic properties differ from the properties of languages that have already been analyzed in previous word segmentation studies.

The ability to segment and recognize unfamiliar words from fluent speech was first explored in the pioneering research developed by P. W. Jusczyk and R. N. Aslin in 1995. In their seminal paper they showed that 7½ -month-old, but not 6-month-old English-learning infants, were able to extract short, monosyllabic word-forms from natural speech passages containing repetitions of two different target words (Jusczyk and Aslin, 1995). Whether familiarized to lists of words and then tested with passages, or familiarized to passages and then tested on words, infants in both testing conditions showed the capacity to extract and recognize possible “lexical” units (word-forms) and they did so by retaining rather detailed information about the phonetic form of these word candidates. Even though words in that experiment were short, simple monosyllabic items (*bike*, *dog*, *cup*, *feet*), infants younger than 7 months of age did not succeed in the task. Follow-up work explored the ability to segment bi-syllabic words and it was shown that for words following the predominant stress pattern in the language (i.e., the trochaic or strong/weak -SW- stress pattern in the case of English), this ability was also present by 7½ months of age (Jusczyk et al., 1999a). Taken together these results were interpreted as an indication that prosodic information, here based on the predominant stress pattern of content words in English (around 90% of content words begin with a stressed syllable, according to Cutler and Carter, 1987), could be used by infants to successfully find word-form units in connected speech. This prosodic hypothesis (defined as the Metrical Segmentation Strategy -MSS- in Jusczyk, 1999) could explain both the results from the monosyllabic and the trochaic word segmentation experiments, as items in the monosyllabic study were strong syllables with full vowels. The importance of prosodic information in early word segmentation was first described in these early studies and subsequent work contributed to give support to the relevant role of prosody in infants' dealing with the word segmentation problem (Johnson and Jusczyk, 2001; Johnson and Seidl, 2008, but see Thiessen and Saffran, 2003; Pelucchi et al., 2009 for an alternative position to the prosodic bootstrapping approach).

If we admit that segmentation strategies based on prosodic information derive from the specific rhythmic properties of the native language, then these strategies might differ in populations acquiring languages with different rhythmic structure. Research with young infants has shown that they are sensitive to global prosodic features contained in the linguistic input (Bosch and Sebastián-Gallés, 1997; Nazzi et al., 2000a). These prosodic features may offer first cues to segment the input into linguistically relevant units such as clauses and phrases within which word-form units can eventually be extracted (Hirsh-Pasek et al., 1987; Nazzi et al., 2000b; Soderstrom et al., 2003; Seidl, 2007; Seidl and Cristià, 2008). But beyond these global prosodic cues, attention to the specific rhythmic properties of the native language and detection of the specific rhythmic unit operating in that language can lead to the emergence of segmentation strategies most adequate to extract words from fluent speech. This is actually the hypothesis behind the so-called *early rhythmic segmentation proposal* developed by Nazzi et al. (2006). Cross-linguistic differences regarding the type of rhythmic strategy and rhythmic unit used for segmentation can then be expected for languages differing in their rhythmic properties.

A gross partition of the languages based on linguistic rhythm has traditionally identified three broad rhythmic types, i.e., stress-timed, syllable-timed and mora-timed languages, each of them associated to a different underlying rhythmic unit (Abercrombie, 1967; Ladefoged, 1975). Germanic languages such as English, Dutch, or German would belong to the first type, having the trochaic stress unit at the basis of their rhythmic structure; Romance languages such as French, Italian or Spanish would be examples of the second type, having the syllable as the basic rhythmic unit and, finally, languages like Japanese would belong to the third type relying on the sub-syllabic *mora* as the basic unit of rhythm. Initially, these typologies were considered to derive from the notion of isochrony between successive units (syllables, feet or morae depending on the type of language), however, subsequent measurements obtained from different languages questioned this idea. Linguistic rhythm is more accurately described as an alternation of elements: vowels and consonants at the most basic level and syllables (stressed and unstressed) and feet at subsequent levels (Nespor et al., 2011). Factors such as variability in syllable structure complexity and the degree of vowel reduction are considered key elements in accounting for language rhythm differences. The study of durational correlates of such phonological phenomena has become the focus of research aimed at identifying the specific properties underlying rhythmic differences between languages.

Different rhythm metrics have been used in studies analyzing limited sets of cross-linguistic material. Ramus et al. (1999) measured duration of vocalic and consonantal intervals in the speech signal. By plotting the percentage of total utterance duration comprising vocalic intervals (%V) against the standard deviation of consonantal intervals (ΔC), they succeeded at adequately grouping the eight languages under study (English, Dutch, Polish, Spanish, Italian, French, Catalan, and Japanese) into the three traditional rhythm typologies. Low et al. (2000) proposed pairwise variability indices (nPVI and rPVI, normalized and raw, respectively) in an attempt to better capture the durational differences between successive vocalic and consonantal intervals. However, only measurements from the nPVI-V scores could group separately English, German, and Dutch on the one hand, and Spanish and French on the other, failing to place Japanese in a different area. Interestingly, languages considered more difficult to classify in terms of rhythm structure, such as Catalan and Polish (Nespor, 1990), showed intermediate positions in the PVI space. More recently, White and Mattys (2007) using rate-normalized metrics of vocalic interval variation (VarcoV) plotted against %V measurements, showed again that “stress-timed” Dutch and English, and “syllable-timed” French and Spanish could be distinguished, but at the same time their analysis revealed that the notion of a strictly categorical distinction between these two rhythmic typologies was far from perfect, with Dutch and French placed in a more intermediate position between stress-timed English and syllable-timed Spanish. In general, results from these metrical studies offer empirical support for the existence of broad rhythmic distinctions between languages, but critically, they also provide a more nuanced perspective on the nature of rhythmic differences that goes beyond the initial notion of three distinct language typologies (see White et al., 2012). From this perspective, differences in the emergence of the word segmentation ability may be found not only when comparing languages traditionally ascribed to a different rhythmic typology (e.g., stress-timed English and syllable-timed French), but also for languages traditionally grouped under the same typology (e.g., syllable-timed French and Spanish, or stress-timed English and Dutch).

What evidence can be found about cross-linguistic differences in the skill to segment words from fluent speech early in development? A review of the early word segmentation literature immediately reveals that research has been developed mostly in English and cross-linguistic data are still scarce. As already mentioned first evidence of word segmentation with natural language material came from English-learning 7½ -month-old infants and restricted to specific types of words such as monosyllabic and trochaic items (Jusczyk and Aslin, 1995; Jusczyk et al., 1999a). Evidence for this ability at an earlier age (6 months) was later attested by using a slightly different methodological approach, in which highly familiar words (infants' own names) preceded the target monosyllabic units (those used in Jusczyk and Aslin, 1995 study) in familiarization passages (Bortfeld et al., 2005). In this situation, familiar words were probably acting as anchors and facilitated segmentation of the adjacent elements, which could not otherwise be easily extracted. Without additional cues to segmentation, English-learning infants are just beginning to segment simple word forms from fluent speech around 7 months of age. It is interesting to note that evidence of segmentation is shown by a familiarity preference pattern, whether familiarization be based on passages or word lists. The direction of the preference has been linked to task demands (Hunter and Ames, 1988). The specific direction of the preference pattern in word segmentation tasks (novelty versus familiarity) and its changes during development can be explained from factors such as the duration of the familiarization, stimulus complexity, degree of similarity between familiarization and test stimuli, and more generally, from expertise acquired with age (Thiessen et al., 2005). Thus at younger ages, when segmentation ability is just emerging, a familiarity preference is to be expected, as found by Jusczyk and Aslin (1995) in 7½ month-old infants.

For studies examining the early emergence of segmentation ability in stress-timed languages other than English, only some data from German and Dutch are available. Segmentation of unstressed closed-class elements has been shown in German-learning infants from 7½ months on, but not before (Höhle and Weissenborn, 2003). The procedure involved familiarization with isolated words and test on passages and, as expected, a familiarity preference was found paralleling Jusczyk and Aslin's results, but this time on unstressed material (although from an acoustical perspective, closed-class grammatical morphemes experience less vowel reduction in German than in English). According to the rhythmic segmentation hypothesis these unstressed monosyllabic elements should have been difficult to segment at that age. It was argued, however, that their special status and potential role in the acquisition of morpho-syntactic knowledge might have favored successful segmentation at an early age.

Evidence from Dutch-learning-infants revealed a slightly later emergence of the segmentation ability (at 9, but not at 7 months of age) using HPP and trochaic words (Kuijpers et al., 1998). Further research replicated 9-month-olds' segmentation of trochees in this language and confirmed that the ability to extract words from fluent speech is not dependent on familiarity with the phonetic structure of the input, as English-learning infants also succeeded in the task with Dutch material (Houston et al., 2000). The same strategy could be exported to successfully extract word units in another stress-timed language with similar rhythmic properties. In spite of the slightly older age of the Dutch participants, segmentation evidence resulted from a familiarity preference. Could rhythmic differences between English and Dutch, as described by White and Mattys (2007) using VarcoV and %V measurements, have impacted speed of segmentation? This remains an open question that deserves further analysis. Unfortunately, no data from monosyllabic word segmentation in Dutch are available, which might have revealed successful segmentation at an earlier age than that obtained for trochees.

The above mentioned studies involve languages traditionally grouped under the stress-timed category, whose rhythmic unit is not the syllable. Will monosyllabic word segmentation be facilitated early in development if the rhythmic unit of the ambient language is the syllable? And will segmentation of bi-syllabic words initially be delayed, being first segmented as two independent syllabic units and only later as whole units? Infant segmentation data from syllable-timed languages are actually limited to French, although evidence obtained from two different French dialects (European and Canadian) is available.

Monosyllabic word segmentation in French has not been extensively explored and only data available from a dissertation indicate that Parisian 7½ month-olds could successfully segment monosyllabic CVC items, using HPP with familiarization to words and test on passages (Gout, 2001). A familiarity preference was also obtained there^1^. No data from infants tested at a younger age were gathered, so we do not know if monosyllabic words in a syllable-timed language are actually easier to extract from fluent speech than similar words in stress-timed languages. What we actually know, however, is that bi-syllabic word segmentation in French is not easily attained, at least according to data from infants exposed to the European French dialect who could only succeed at successfully segmenting iambs by 16 months of age (Nazzi et al., 2006). Data from French-learning infants exposed to the Canadian dialect did not replicate European French results, however. No “delayed” segmentation ability was identified in Canadian French-learning infants compared to a group of Canadian English young learners tested at 8 months on two-syllable word segmentation (iambic and trochaic patterns, respectively): both groups succeeded, although segmentation strategies certainly differed and were adjusted to the properties of the native language, so no group was able to segment the items in the other language (Polka and Sundara, 2012).

Because Nazzi et al.'s (2006) and Polka and Sundara's (2012) work involved a considerable amount of experiments to more thoroughly explore segmentation abilities in the populations under study, some relevant findings about the segmentation of the syllabic components of the iambic items could be identified. Clear evidence of final syllable segmentation was obtained at 12 months and some evidence of initial syllable segmentation could also be found at the same age in European French infants suggesting that a syllable-based segmentation procedure is applied before bi-syllabic words can be successfully segmented as whole units (Nazzi et al., 2006). Similarly, although at an earlier age, results in Canadian French also revealed some ability to segment each isolated syllable of the iambic target words, although the transition from an initial syllable-based segmentation to a successful whole bi-syllabic word segmentation could not be established in that research as only groups of 8-month-olds' were tested (Polka and Sundara, 2012). Relevant for our own research on monosyllabic word segmentation, the Canadian study found opposite response patterns when familiarization involved whole iambic words or only their syllabic components. The novelty preference pattern obtained when syllables instead of whole words were presented in the familiarization phase suggests that syllables might be more easily identified because they match the rhythmic unit in this language.

Taken together, and compared to data from segmentation in stress-timed languages, research done in French reveals important cross-linguistic differences, not only in the emergence of segmentation abilities, but also in the strategies used, which reflect the rhythmic nature of the language of exposure. However, French results are not clear-cut especially due to the non-trivial timing difference in the emergence of segmentation abilities found when both dialects are compared. Even if differences can be attributed to factors derived from specific properties of these dialects or the testing material, the fact is that behavioral results so far have only partially confirmed a hypothetic ease to segment monosyllabic words or track syllabic elements in fluent speech, as it could be expected if the syllable is the rhythmic unit for segmentation in syllable-based languages (but see Goyet et al., 2010 for a re-assessment of syllabic segmentation using ERP measures). Studying early segmentation abilities in infants acquiring other syllable-timed languages could shed more light on the early rhythmic segmentation hypothesis and help clarify results obtained so far.

Spanish and Catalan have also been traditionally grouped under the syllable-timed typology, although some metric distinctions have been described in studies comparing the rhythmic properties of these two languages. Some authors consider Catalan a rhythmically-intermediate language between the stress-timed and syllable-timed typologies (Nespor, 1990). Catalan, but not Spanish, has vowel reduction, a property that can affect syllabic rhythm and determines differences in the type of vowels that can appear in unstressed syllable positions (Prieto et al., 2012). Catalan allows for more complex consonant clusters in coda position, while syllabic structures are simpler in Spanish. As a consequence, %V metrics have been found to be significantly lower in Catalan than in Spanish. However, higher variability in vocalic interval duration (i.e., higher VarcoV scores that characterize languages with vowel reduction) has not been confirmed, with Catalan even showing lower variability scores than Spanish according to Payne et al.'s (2009) work. More recent research has corroborated that vowel reduction in Catalan does not seem to substantially increase variability in vowel interval duration (Prieto et al., 2012). In sum, while some rhythmic differences between Catalan and Spanish exist, the classification of Catalan as a rhythmically-intermediate language between syllable-timed and stress-timed typologies remains controversial. Although an in-depth and systematic comparison between Spanish, Catalan, and French rhythm metrics is not available, measures from different studies involving different sets of material would suggest a non-overlapping distribution of these three “syllable-timed” languages over the %V and VarcoV rhythmic plane (White and Mattys, 2007; Payne et al., 2009). Among these three languages, Spanish would show the highest %V and the lowest VarcoV scores, while French would show the opposite tendency (i.e., higher VarcoV and lower %V scores), and Catalan would be placed in an intermediate position, probably more similar to Spanish in terms of vocalic interval variability (VarcoV), as the above mentioned studies have revealed. Given these differential metrical characteristics, Spanish and Catalan are good language candidates to extend word segmentation studies in syllable-timed languages other than French and explore infants' early use of a syllabic segmentation strategy. In particular, the comparison between Catalan-learning and Spanish-learning groups can reveal if the (minor) rhythmic differences between these two languages have an impact on the emergence of the segmentation ability.

To sum up, the present research was designed to explore the emergent ability to segment monosyllabic word-forms by infants acquiring Spanish, Catalan, but also both languages simultaneously from birth. To our knowledge, word segmentation abilities in bilingual infants have begun to be explored only in English-French environments, with preliminary data available so far showing bi-syllabic word segmentation ability in both languages by 8 months of age (Polka and Sundara, 2003). The inclusion of bilingual participants in this research, exposed to languages traditionally grouped into the same rhythmic class, but nonetheless showing some minor differential rhythmic properties, can contribute to clarify the actual impact that bilingual exposure can have on the emergent ability to extract words from connected speech, when segmentation strategies derived from each of the ambient languages are likely to converge.

In the present research, evidence of an emergent segmentation ability will be explored using the HPP technique, in line with the work just reviewed coming from both stress-timed and syllable-timed languages. However, we have selected the less frequent order in this type of experiments, involving passages first and test on lists of isolated words. Because similar segmentation effects were obtained independently of the testing order in the original Jusczyk and Aslin's (1995) study, we opted for the passages-first order to promote segmentation spontaneously arising from a more natural context and to avoid initially biasing participants to attend to a specific word or syllabic unit.

In our first experiment we analyzed 8-month-olds' ability to segment words that match the rhythmic unit of their native language. No great difficulties were expected for monosyllabic word segmentation in our Catalan and Spanish participants, but given the limited data available in French and the slightly delayed emergence of the segmentation ability, even for the syllabic components of the bi-syllabic words, found by Nazzi et al. (2006), evidence from the three groups tested at 8 months would be most informative about the timing of this emergent ability in languages different from French but having syllables as the basic rhythmic units.

In a second experiment we wanted to further explore if evidence of monosyllabic word segmentation could be found at an earlier age (6 months) in syllable-timed languages compared to stress-timed ones, due to the direct match between the target elements (monosyllabic words) and the rhythmic unit for segmentation (the syllable). If confirmed, results would not only give support to the early rhythmic segmentation hypothesis, but they would also suggest the need to take into account additional differences in the rhythmic properties of languages traditionally grouped into the same rhythmic typology, as these properties might lead to differences in the timing of the emergence of the segmentation ability. Recall that the earliest evidence for monosyllabic word segmentation in French comes from a single experiment with 7½-month-olds showing a familiarity preference response pattern (Gout, 2001; Gout, unpublished dissertation).

The ultimate aim of the present study is to set the groundwork for future research exploring the emergence of the ability to segment multi-syllabic word-forms both in Spanish- and in Catalan-learning infants. Knowledge about the ability and the segmentation strategies used to extract short, simple monosyllabic units from connected speech can offer valuable information to better understand the specific problems that segmenting bi- and tri-syllabic words in syllable-timed languages with variable stress can pose to the infant learner.

## Experiment 1: word segmentation at 8 months

### Participants

A total of 54 healthy full-term infants with no history of hearing or vision problems were included in the sample divided into three groups (*N* = 18 in each group) according to the language/s spoken in their environment (Catalan only, Spanish only or both languages on a daily basis). Mean age of the infants in the Catalan monolingual group was 8 months 4 days (range: 7 months, 15 days–8 months, 22 days); in the Spanish monolingual group was 8 months 6 days (range: 7 months, 19 days–8 months, 25 days) and in the bilingual group was 8 months 6 days (range: 7 months, 13 days–8 months, 15 days). No significant between-group age differences were found (*F* < 1). Participants were assigned to different language groups based on the information obtained through a questionnaire to the parents that offered an estimate of the daily and weekly amount of exposure to the languages in their environment (Bosch and Sebastián-Gallés, 2001). To be included in a monolingual group, participants had at least 75% of regular exposure to either Catalan or Spanish, while a more balanced distribution between these two languages was required for inclusion in the bilingual group. Mean percentage of exposure to Catalan in the Catalan monolingual group was 92% (range: 75–100%) and to Spanish in the Spanish monolingual group was 93% (range: 80–100%). From the 18 infants in the bilingual group, seven had a higher amount of exposure to Spanish than to Catalan (66–34%) and they were tested on Spanish material. The remaining infants had a higher exposure to Catalan than to Spanish (63–37%) and were tested on Catalan material. Fourteen additional infants were also tested but excluded from the final sample due to fussiness or crying leading to incomplete testing (4), very short looking time—below 1 s—to trials in the test phase (6), preterm birth (1) and experimental error (3).

### Stimuli

Target Spanish and Catalan monosyllabic words with full vowels and a CVC (*bus*, *mar*, *gol* –“bus,” “sea,” and “goal”-) or CCVC (*tren* –“train”) structure were selected because of their cognate status in the languages under study (for simplicity, from now on we will refer to all target words as having a monosyllabic CVC structure). Target words were nouns that are infrequent in the first receptive and expressive vocabularies of 1-year-olds acquiring Spanish, Catalan or both (Águila et al., 2005).

Four passages were created, formed by six different sentences each with the target word appearing once per sentence in different positions (twice in initial, twice in medial and twice in final sentence positions). Because the experimental design involved two different conditions (half of the participants were familiarized with “train-bus” passages -TB-, and the other half with “gol-mar” passages -GM-), parallel sentences were used to make conditions equivalent (see Table 1). Adjacent syllables to the target words (from words preceding or following the target nouns) were controlled so that no specific syllabic sequences appeared repeatedly within the passage. Mean duration of the sentences in the passages was 2.3 s and total length of the passages was adjusted to 18 s by inserting short pauses of about 700 ms between sentences. Passages had 45–46 syllables each and especial care was taken to build equivalent passages for the Spanish and Catalan versions of the material. Sentences were not always perfect translations because length of the words tends to be shorter in Catalan and we wanted to keep with the same number of syllables per sentence. In spite of minor meaning differences between Spanish and Catalan sentences (irrelevant to study word segmentation in early infancy), the final passages represent equivalent versions of the material in terms of number of syllables and total length.

**Table 1:** **Catalan and Spanish sentences forming the passages used in the familiarization phase**.

**“Tren” passage (*train*)**
*Catalan:* Un tren té sis o set vagons. Veig un gran tren des d'aquí. El tren no s'atura mai. A la foto hi ha aquell tren. Mira aquest cotxe a prop del tren. Arriben en tren molt d'hora
*Spanish:* Un tren tiene seis vagones. Veo un gran tren desde aquí. El tren nunca está parado. En la foto está aquel tren. Mira este coche junto al tren. Llegan en tren mañana
**“Bus” passage (*bus*)**
*Catalan:* Un bus va venir de sobte. Esperava el primer bus. Recordo aquest bus cada dia. El bus no era massa bo. M'encanta el seu bus de cartró. Somiaré amb el meu bus
*Spanish:* Un bus llega de repente. Esperan otro bus. Recuerdo aquel bus cada día. El bus no era largo. Me encanta su bus de cartón. Soñaré con este bus
**“Mar” passage (*sea*)**
*Catalan:* Un mar té milers de peixos. Veig un gran mar des d'aquí. El mar no s'atura mai. A la foto hi ha aquell mar. Mira aquest cotxe a prop del mar. Arriben per mar molt d'hora
*Spanish:* Un mar tiene muchos peces. Veo un gran mar desde aquí. El mar nunca está calmado. En la foto está aquel mar. Mira este coche junto al mar. Llegan por mar mañana
**“Gol” passage (*goal*)**
*Catalan:* Un gol va venir de sobte. Esperava el primer gol. Recordo aquest gol cada dia. El gol no era massa bo. M'encanta el seu gol de taló. Somiaré amb el meu gol
*Spanish:* Un gol llega de repente. Esperan otro gol. Recuerdo aquel gol cada día. El gol no era bueno. Me encanta su gol de tacón. Soñaré con este gol

In the experimental design half of the participants were familiarized to “Tren-Bus” (train-bus) passages and the other half to “Mar-Gol” (sea-goal) passages.

Word lists involving 12 isolated productions of each of the four target words were also needed for use in the test phase of the experiments. Six different tokens of the same noun repeated twice in a randomized order formed each of the four experimental word lists in this study. Total length of the word lists was18 s as lists were built by adding silence to the end of the stimulus to reach a 1.5 s duration (mean length of the words in the lists in each language is reported in Table 2).

**Table 2:** **Acoustic measures of target words in passages (familiarization) and lists (test) for Catalan and Spanish material**.

	**Passage words**	**List words**	
	**Mean (*SD*); range**	**Mean (*SD*); range**	***p***
**DURATION (ms)**			
Catalan	380 (43.4); 331–488	596 (78.2); 474–763	^***^
Spanish	378 (59.5); 290–536	599 (120); 422–863	^***^
*p*	n.s.	n.s.	
**AMPLITUDE (dB)**			
Catalan	71.9 (4.2); 65.4–80	70.5 (1.2); 68.2–72.5	n.s.
Spanish	72.9 (3.1); 68.4–78.4	71.4 (3.3); 61.2–77.2	n.s.
*p*	n.s.	n.s.	
**PITCH (Hz)**			
Catalan	256 (61); 174–388	267 (40); 201–372	n.s.
Spanish	274 (57); 194–396	283 (56); 201–406	n.s.
*p*	n.s.	n.s.	

Measurements include whole word duration (ms), followed by amplitude (dB) and pitch (Hz) calculated on the vocalic portion of the words. Significant differences are also indicated.

Results of t_(23)_ tests:

*** p < 0.001; n.s. p > 0.05.

Passages and words were produced by a highly proficient Spanish-Catalan bilingual female speaker and she was instructed to use infant direct speech, as if speaking to a young child. The stimuli were recorded in a single session in a comfortable, sound attenuated booth equipped with an omni-directional microphone. Utterances were recorded directly onto a Pentium- III PC using Sound Edit (version 2.99) software. The operating system was Windows XP. Online monitoring ensured optimal sound quality recording.

Finally, to ensure similarity between the materials for each language, acoustic analyses on target words extracted from the passages and words in the lists were conducted using Praat software (version 5.3.22). Mean values of word duration and amplitude (for the entire word) and pitch (calculated on the vocalic portion of the word) were obtained and are reported in Table 2, both for Catalan and Spanish material. As expected, statistical analyses only revealed significant differences in duration between words extracted from the passages and words produced in isolation. Amplitude and pitch measurements were found equivalent both within each language and also between languages (see details in Table 2).

### Procedure

The familiarization-preference procedure with familiarization to passages and test on lists of words [as in Experiment 4, by Jusczyk and Aslin (1995)], was implemented in this research. The testing took place in a three-sided test booth, but instead of a frontal and two lateral lights typically used in the HPP set-up, a frontal display involving three computer screens and two concealed loudspeakers below the left and right monitor screens was used [this set-up had already been satisfactorily used by Bosch and Sebastián-Gallés (2001), to test for language discrimination in young infants]. Babies were seated on their parent's lap facing these three frontal monitor screens. Parents were listening to music through headphones throughout the whole experimental session. An experimenter, inside the testing room but out of the view of the infant, watched infants' looking behavior through a TV monitor, controlled trial presentation and recorded online infants' attention. By pressing and releasing the mouse button the experimenter could register the direction and duration of the infant look fixation toward the side screen involved in the presentation of the audio files in each trial. Online information about total attention time in each trial for each participant was stored and could later be checked against the results from off-line coding of the recordings to assess reliability of the measures and detect experimenter errors.

The experimental session began with a familiarization phase in which TB or GM passages were presented on alternating trials until the infant accumulated 45 s of attention time to each passage. Because of this criterion, infants could hear the target words in sentential contexts about 18 times each. Immediately after completing familiarization, the test phase began. It involved 16 test trials (four target word lists presented in four blocks). Words within each list were randomly presented and the order varied for each participant. At the beginning of each trial the central monitor displayed a flashing green circle to direct infants' attention toward the center. Immediately afterwards, one of the two lateral monitors displayed a flashing red circle to capture infant's attention and as soon as the infant oriented toward that side screen the audio files were presented. Auditory material was played until trial completion (18 s) or until the infant ceased to look in that direction for more than two consecutive seconds. In case of trial interruption, passage presentation was not resumed in the next trial, but started again from the beginning. Looks away below 2 s duration did not interrupt trial presentation but time away was not included in the final amount of fixation for that specific trial.

### Design

Half of the infants were familiarized to passages containing the target nouns “tren-bus” (TB condition) and the other half to passages containing the target nouns “gol-mar” (GM condition). In the test phase all participants were presented with the four target word lists.

## Results

Separate analyses were run, one on changes in attention time from the first to the last trial in the familiarization phase, and the other on attention time to familiar vs. novel words in the test phase. Regarding changes in attention during familiarization to the passages, a repeated-measures ANOVA with looking time as dependent measure, language group (Catalan, Spanish, bilingual) and familiarization condition (TB or GM) as a between-group factors and trial (first, last) as repeated measures revealed a highly significant effect of familiarization trial [*F*_(1, 48)_ = 82.9; *p* = 0.0001; η^2^= 0.63], but no effect of language group or condition and no significant interactions (all *F'*s < 1). All three groups thus showed similar decays in attention from the first to the last trial in the familiarization phase when they were presented with the passages containing repetitions of two target words (mean attention time to first and last trial was, respectively, 15.1 s and 9.9 s in the Catalan monolingual group, 16 s and 8.2 s in the Spanish monolingual group and 15.2 s and 10.5 s in the bilingual group). Paired *t* tests conducted separately for each group on attention time to first and last familiarization trial confirmed the similarity in behavior [Spanish monolingual: *t*_(17)_ = 6.1, *p* = 0.0001; Cohen's *d* = 2.0]; [Catalan monolingual: *t*_(17)_ = 4.7, *p* = 0.0001; Cohen's *d* = 1.41]; [bilingual: *t*_(17)_ = 4.9, *p* = 0.0001; Cohen's *d* = 1.21].

We also analyzed if groups differed in the number of trials to reach criterion. A one-way ANOVA on number of trials in the familiarization phase as dependent measure and condition (TB vs. GM) and language group (Spanish, Catalan and bilingual) as between-subjects factors revealed no significant effects (*F'*s < 1) or interaction [*F*_(2, 48)_ = 1.77, *p* = 0.18; η^2^ = 0.69]. These results suggest that duration of the familiarization and participants' looking behavior in this phase can be considered equivalent.

**Figure 1:**
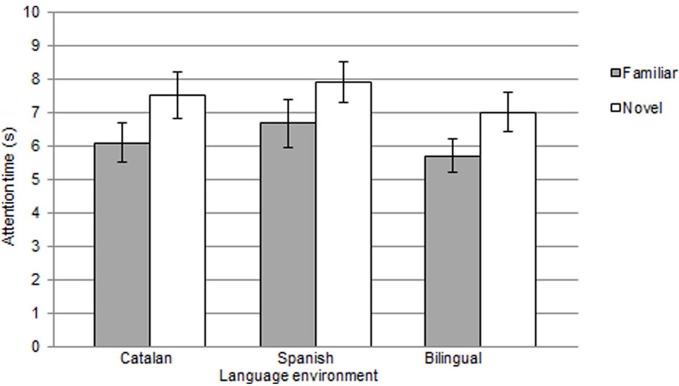
**Mean attention time (s) and standard error to familiar and novel words presented in the test phase, for the 8-month-old infants, grouped by language environment (monolingual Catalan, monolingual Spanish and bilingual)**.

To assess word segmentation, mean attention time to familiar vs. novel words in the test phase was computed for each participant (see Figure 1). A repeated-measures ANOVA with mean looking time as dependent measure, language group (Catalan, Spanish, bilingual) and familiarization condition (TB or GM) as a between-group factors and type of word (familiar, novel) as repeated measures was run. Results only revealed a highly significant main effect of type of word [*F*_(1, 48)_ = 21.6; *p* = 0.0001; η^2^ = 0.31], with no language group or condition effects (both *F's* < 1) and no interactions. Paired *t* tests conducted separately for each group on mean attention time to familiar vs. novel word lists confirmed the presence of significant differences in attention to the two types of words, thus indicating that segmentation of monosyllabic words had been reached [Spanish monolingual: familiar words *M* = 6.6 s (*SD* = 3.2) and novel words *M* = 7.9 s (*SD* = 2.7); *t*_(17)_ = −2.7, *p* = 0.015; Cohen's *d* = 0.41]; [Catalan monolingual: familiar words *M* = 6.1 s (*SD* = 2.8) and novel words *M* = 7.5 s (*SD* = 3); *t*_(17)_ = −2.6, *p* = 0.019; Cohen's *d* = 0.48]; [bilingual: familiar words *M* = 5.7 s (*SD* = 2.3) and novel words *M* = 7 s (*SD* = 2.7); *t*_(17)_ = −2.8, *p* = 0.011; Cohen's *d* = 0.51]. Interestingly, however, the pattern of preference that was obtained at 8 months across all three groups was not the expected one, as a familiarity preference rather than novelty is typically observed in segmentation tasks using natural language. The monosyllabic nature of the target items, the fact that they were presented twice in sentence-final position in the familiarization passages, together with the use of IDS and a sufficiently long familiarization phase are possible factors that might explain this unexpected novelty preference, which is more likely to be found when the task is relatively easy and can be completed within the temporal limits established by the procedure.

## Experiment 2: word segmentation at 6 months

### Participants

As in Experiment 1, a total of 54 healthy full-term infants with no history of hearing or vision problems were included in the sample divided into three groups (*N* = 18 in each group) according to the language/s spoken in their environment (Catalan only, Spanish only or both languages on a daily basis). Mean age of the infants in the Catalan groups was 6 months 6 days (range: 5 months, 22 days–6 months, 29 days); in the Spanish monolingual group was 6 months 4 days (range: 5 months, 19 days–6 months, 27 days) and in the bilingual group was 6 months 7 days (range: 5 months, 19 days–6 months, 27 days). No significant between-group age differences were found (*F* < 1). Following the information from the initial language questionnaire to parents, participants were assigned to different language groups. Inclusion criteria were the same as in Experiment 1. Mean percentage of exposure to Catalan in the Catalan monolingual group was 91% (range: 80–100%) and to Spanish in the Spanish monolingual group was 95% (range: 75–100%). From the eighteen infants in the bilingual group, 12 had a higher amount of exposure to Spanish than to Catalan (65–35%) and they were tested on Spanish material. The remaining six had a higher exposure to Catalan than to Spanish (64–36%) and they were tested on Catalan material. Twenty-nine additional infants were also tested but excluded from the final sample due to fussiness or crying leading to incomplete testing (22), very short looking time—below 1 s—to trials in the test phase (5) and experimental error (2).

### Stimuli, procedure, and design

Same as in Experiment 1.

## Results

Separate analyses were also run on data from these younger-age groups to explore attention behavior in the familiarization phase (expected decay of looking time) and possible differences in attention time to familiar vs. novel words in the test phase, as indicative of successful word segmentation.

Concerning attention behavior during familiarization, a repeated-measures ANOVA with looking time as dependent measure, language group (Catalan, Spanish, bilingual) and familiarization condition (TB or GM) as a between-group factors and trial (first, last) as repeated measures revealed a highly significant effect of familiarization trial [*F*_(1, 48)_ = 42.9; *p* = 0.0001; η^2^ = 0.47], and no effect of language group or condition and no significant interactions [familiarization trial × language group: *F*_(2, 48)_ = 1.15; *p* = 0.32; η^2^ = 0.04; familiarization trial × condition: *F*_(1, 48)_ = 1.7; *p* = 0.19; η^2^ = 0.03; familiarization trial × language group × condition: *F* < 1]. All three groups showed a decrement in their attention time during familiarization to passages containing repetitions of target words (mean attention time to first and last trial was, respectively, 15.5 s and 12.3 s in the Catalan monolingual group, 15.5 s and 10.6 s in the Spanish monolingual group and 14.9 s and 9.1 s in the bilingual group). Paired *t* tests conducted separately for each group on attention time to first and last familiarization trial confirmed the similarity in this behavior [Spanish monolingual: *t*_(17)_ = 4.1, *p* = 0.001; Cohen's *d* = 1.24]; [Catalan monolingual: *t*_(17)_ = 2.9, *p* = 0.009; Cohen's *d* = 0.85]; [bilingual: *t*_(17)_ = 4.2, *p* = 0.001; Cohen's *d* = 1.29]. We also analyzed if groups differed in the number of trials to reach criterion. A One-Way ANOVA on number of trials in the familiarization phase as dependent measure and condition (TB vs. GM) and language group (Spanish, Catalan, and bilingual) as between-subjects factors revealed no significant effect of language group (*F* < 1), but a significant effect of condition [*F*_(1, 48)_ = 5.59, *p* = 0.02; η^2^ = 0.1], with no significant group × condition interaction [*F*_(2, 48)_ = 1.11, *p* = 0.33; η^2^= 0.04]. Follow-up *t* tests revealed that mean number of trials to reach criterion in the TB condition (9.1) was significantly higher than in the GM condition (7.5) [*t*_(26)_ = −2.56, *p* = 0.017; Cohen's *d* = 0.67]. Further *t* tests by language groups indicated that only in the monolingual Catalan group the number of trials to reach criterion was significantly different by condition [*t*_(8)_ = −2.8, *p* = 0.02; Cohen's *d* = 1.5]. Differences by condition did not reach significance in the other two groups [monolingual Spanish: *t*_(8)_ = −1.04, *p* = 0.32; Cohen's *d* = 0.26; bilingual group *t* < 1].

**Figure 2:**
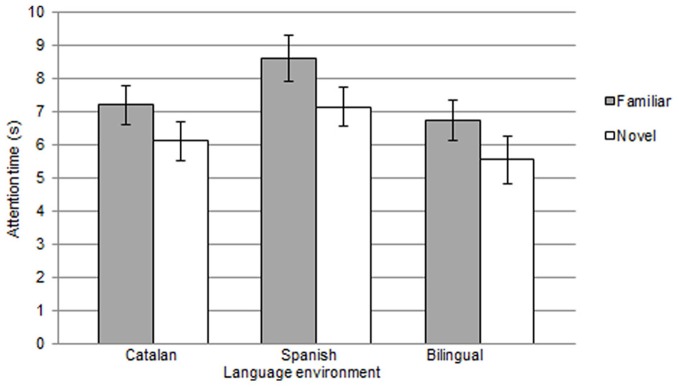
**Mean attention time (s) and standard error to familiar and novel words presented in the test phase, for the 6-month-old infants, grouped by language environment (monolingual Catalan, monolingual Spanish and bilingual)**.

To analyze word segmentation ability in the younger groups, mean attention time to familiar vs. novel words in the test phase was computed for each participant (see Figure 2). A repeated-measures ANOVA with mean looking time as dependent measure, language group (Catalan, Spanish, bilingual) and familiarization condition (TB or GM) as a between-group factors and type of word (familiar, novel) as repeated measures was run. Results only revealed a highly significant main effect of type of word [*F*_(1, 48)_ = 18.9; *p* = 0.0001; η^2^ = 0.87], with no language group or condition effects [language group: *F*_(2, 48)_ = 1.9; *p* = 0.15; η^2^ = 0.07; and condition *F* < 1] and no significant interactions (*F'*s < 1). Paired *t* tests conducted separately for each group on mean attention time to familiar vs. novel word lists confirmed the presence of significant differences in attention to the two types of words, thus indicating that segmentation of monosyllabic words had successfully been reached at this early age [Spanish monolingual: familiar words *M* = 8.6 s (*SD* = 3.1) and novel words *M* = 7.1 s (*SD* = 2.7); *t*_(17)_ = 2.2, *p* = 0.035; Cohen's *d* = 0.48]; [Catalan monolingual: familiar words *M* = 7.1 s (*SD* = 2.8) and novel words *M* = 6.1 s (*SD* = 2.7); *t*_(17)_ = 4.3, *p* = 0.0001; Cohen's *d* = 0.39]; [bilingual: familiar words *M* = 6.7 s (*SD* = 2.8) and novel words *M* = 5.5 s (*SD* = 2.9); *t*_(17)_ = 2.2, *p* = 0.038; Cohen's *d* = 0.41]. Overall, results indicate that 6 month olds (monolinguals and bilinguals) can segment monosyllabic words from sentential contexts and evidence for this ability is reflected in the familiarity preference response pattern observed for words in the test phase. This is actually the usual preference pattern obtained in this type of task and it differs from the pattern found with the older groups tested in this research using exactly the same material and procedure.

A final analysis involving data from the two age groups was undertaken and only the age (6 vs. 8 months) per type of list (familiar vs. novel) interaction was deemed significant [*F*_(1, 102)_ = 40.4; *p* = 0.0001; η^2^ = 0.28], confirming the radical change in the direction of preference that had taken place between the two ages under analysis. No other effects or interactions were found significant in this global analysis. We also extended the analysis to the attention time measures in the familiarization phase to check for any between-age differences in attention behavior to trials in the familiarization that could be related to the word preferences observed in the test phase. Results yielded no evidence of significant differences by age related to the attention time measures in the familiarization phase [*F*_(1, 102)_ = 1.55, *p* = 0.21; η^2^ = 0.01], nor in the number of trials to reach criterion [*F*_(1, 107)_ = 1.34, *p* = 0.24; η^2^ = 0.14]. To sum up, although age differences did not seem to affect the behavior in the familiarization phase, they were determinant in the preference pattern observed in the test.

## Discussion

This research has explored young infants' emerging ability to segment simple, monosyllabic word-forms from fluent speech (natural language) in syllable-timed languages other than French. Six and eight-month-old participants growing up in Catalan monolingual, Spanish monolingual and Spanish-Catalan bilingual families were tested on a version of HPP, with familiarization to passages containing repetitions of two different target words in sentential contexts, and tested on words. Results revealed that all groups at both ages were able to successfully segment words from the passages and recognize them in the test phase. Critically, however, the predominant response pattern obtained differed with age. While younger infants showed a familiarity preference, by far the most frequent pattern found in segmentation studies using natural language paradigms (Jusczyk and Aslin, 1995; Jusczyk et al., 1999a,b; Mattys et al., 1999; Mattys and Jusczyk, 2001a,b; Houston et al., 2004), older groups showed a novelty preference, i.e., a preference for the lists involving words not included in the familiarization passages.

Familiarity or novelty preference patterns are usually attributed to the ease or difficulty to solve the task at hand (Hunter and Ames, 1988). As an example, in segmentation studies using artificial language paradigms evidence of segmentation is usually linked to a novelty preference for part-words over words in the familiarization material. This is no surprise as artificial languages in these studies are considered more simplistic than natural languages, syllabic sequences lacking the higher levels of variability found in any of the relevant dimensions in natural speech material (Pelucchi et al., 2009). Based on language complexity factors, word segmentation experiments run on natural speech material are thus likely to yield results showing a familiarity rather than a novelty preference and this is the pattern that a priori could be expected in our research. However, our study focused on simple elements (monosyllabic words) to be segmented from passages recorded in IDS style and participants were acquiring a language in which the rhythmic unit is the syllable (Nazzi et al., 2006), so even if the experiment was run on natural language material, the sum of all these factors may have contributed to simplify the task, thus leading to a novelty preference pattern in infants' responses. This can especially be true at older ages, when greater ability in word segmentation might have already been acquired.

There are thus a number of factors that may have facilitated word segmentation in the populations under study. The use of IDS in the recording of the material is one of them. IDS has been described as having a slower rate of speech, longer pauses and greater pitch excursions favoring infant's attention to it (Fernald and Kuhl, 1987). This style also uses simplified sentence structures that together with prosodic exaggeration can facilitate speech processing and the extraction of units from the segmentation perspective. Support for this interpretation comes from a segmentation study in which nonsense sentences either with in ADS or IDS style were used to test segmentation ability in 7- and 8-month-old infants (Thiessen et al., 2005). Results indicated that only in the IDS condition segmentation could be reached, thus the prosodic characteristics of IDS seem to have facilitated the extraction of word-form units from material that was otherwise equivalent in terms of the statistical cues that could be used for segmentation. In our material, where sentences in the passages were about 7–8 syllables long and target words where often aligned to phrase boundaries, clearly demarcated by pauses, the extraction of the target elements from the passages was certainly facilitated (Seidl and Johnson, 2006). It is worth mentioning here that words in the passages and words in the lists differed in duration, as reported in Table 2. Variability did not preclude recognition of the target items: infants in any of the two age groups in this study did not fail to notice the correspondence between the target words placed in sentential contexts and the words presented in isolation in the test phase, when duration was longer than when they were produced in sentential contexts. This result is similar to what has already been found in previous research using natural speech (Jusczyk and Aslin, 1995; Jusczyk et al., 1999a; Mattys and Jusczyk, 2001a,b), but it is reported here for infants tested at a younger age (6 months).

Another factor favoring word segmentation in our research is related to the length of the words (monosyllabic CVC items) and the match with the rhythmic unit of the languages under study. This is actually a key issue in our research. From the early rhythmic segmentation hypothesis, syllabic units would play a determinant role at the onset of word segmentation for infants acquiring languages with a syllable-timed rhythm (Nazzi et al., 2006). Thus, segmentation of monosyllabic word-forms should be easier in these languages than in languages belonging to a different rhythmic typology in which the rhythmic unit might not be the syllable. The fact that positive evidence for monosyllabic word segmentation has been obtained at 6 months of age in either Catalan-learning and Spanish-learning infants suggests that the match between the rhythmic unit and the length of the target words in our study may have favored an early onset of the segmentation abilities in our populations. Recall here that English-learning infants succeeded at monosyllabic word segmentation with natural language material at 7½ months of age but not earlier (Jusczyk and Aslin, 1995), unless highly familiar words such as the infants' own names preceded the target monosyllabic units facilitating the extraction of adjacent elements (Bortfeld et al., 2005). Without these or other additional cues to segmentation, English-learning infants seem to start segmenting simple word forms from fluent speech around 7 months of age.

It is interesting to note that in spite of the presence of some differential features between Catalan and Spanish possibly affecting their rhythmic properties (vowel reduction and more complex consonantal codas in the former), these differences have not had any clear impact on the emergence of the segmentation ability for monosyllabic word-forms. Neither the monolingual, nor the bilingual groups in this research have shown significant differences in their behavior in the segmentation task.

Data from other syllable-timed languages that could support the early rhythmic segmentation hypothesis are limited to French and mostly focused on bi-syllabic word segmentation (Nazzi et al., 2006; Goyet et al., 2010 for European French, and Polka and Sundara, 2012, for Canadian French) so no data are available regarding 6-month-old French-learning infants solving a word segmentation task. However, as mentioned in the introduction, these studies have reported a certain ease for syllabic segmentation compared to the segmentation of bi-syllabic words, so in spite of the differences and controversies when European and Canadian French segmentation studies are compared, there is some converging evidence about the facilitative role of the syllable as a unit for segmentation in these syllable-timed languages. But, finding differences between Spanish and Catalan on the one hand, and French on the other, on the early onset of word segmentation for monosyllabic units is also a possibility to be taken into account. Spanish and Catalan have contrastive and variable stress, a property not shared with French. Stress in French falls invariably on the last syllable of each word of phrase but it is actually mostly reduced in fluent speech; prominence of stressed syllables is very similar to their unstressed neighbors and only words in utterance-final position get some prosodic marking in the form of vowel lengthening (Tranel, 1987). The presence of variable stress in languages such as Catalan or Spanish can be a factor that enhances the perception of syllabic units, thus leading to an earlier onset of the segmentation ability at least for short monosyllabic items. This remains an open question requiring further analysis, but the positive effect of variability in the input to the young learner has already been pointed out in research addressing different aspects of language acquisition. For instance, high levels of acoustic/phonetic variability deriving from the use of multiple exemplars (several tokens from multiple speakers) in a word learning task involving phonologically similar words led participants to successful learning while they failed in a more simple, single-exemplar condition (Rost and McMurray, 2009). Another example can be found in research showing that the learning of non-adjacent dependencies is facilitated with decreasing predictability between adjacent elements, that is, the extraction of the invariant structure (the stable elements of a stimulus set) is actually easier with increasing variability of the irrelevant intervening elements (Gómez, 2002). Back to word segmentation in syllable-timed languages, it is possible to hypothesize that the presence of variable stress in the input may have enhanced the detection and extraction of monosyllabic word units. This is an issue to be further analyzed in future studies, where the facilitation effects of the syllable as the rhythmic unit for segmentation could be more carefully analyzed after controlling for other facilitation effects derived from the paradigm, task demands, or the specific properties of the speech material in the test.

The developmental change in the preference pattern obtained in our data, suggests rapid gains in segmentation ability for these short, monosyllabic units that match the rhythmic unit of the ambient language. Because the paradigm and material used in our experiments were exactly the same at both ages, the reversal of the preference pattern seems to confirm the ease to extract these short units from sentential contexts with increasing age. A reversal of the preference pattern had also been described in the literature (Thiessen et al., 2005), but in that case not only age but an extended familiarization phase were both factors that modified the pattern obtained at an earlier age. This is not the case in our study as no manipulation of the paradigm was done. A simpler interpretation is thus that infants have gained expertise in segmenting fluent speech, especially regarding monosyllabic elements. The question remains whether similar results would be obtained for CV items, instead of CVC, and whether segmentation of function CVC or CV words in these languages, involving unstressed vowels, would also be successfully solved at an early age and by all language groups (the presence of vowel reduction in Catalan but not in Spanish may also contribute to differential results). This is clearly a topic to be explored in future research.

The present paper has included participants growing up bilingual and their results deserve some comments. An early onset of monosyllabic word segmentation abilities has also been found in our Spanish-Catalan infant participants. The timing and characteristics of their segmentation ability do not seem to differ from results obtained in monolingual infants in our study, at least from a behavioral perspective. The bilingual results are relevant in that they are the first evidence of segmentation abilities in bilinguals acquiring languages with rather similar rhythmic properties [so far, only preliminary data exist from French-English bilinguals showing bi-syllabic word segmentation ability in both languages by 8 months of age, as reported by Polka and Sundara (2003)]. Although bilinguals in our research are exposed to languages that do not greatly differ in their rhythmic properties and, from this perspective, it could be predicted that no delays or differences in solving the segmentation task would be found, bilingual exposure might nevertheless lead to small differences in the developmental time-course of certain speech and language abilities, as for instance those found in the phonetic categorization domain (Bosch and Sebastián-Gallés, 2003; Sebastián-Gallés and Bosch, 2009). Even if the ambient languages do not show great differences in their rhythmic characteristics, segmentation abilities might have been slightly delayed in this population, just as a consequence of adaptive processes to cope with the more complex input the bilingual is exposed to. This was not the case in our data, with bilinguals showing parallel results to their monolingual counterparts. These data extend to the word segmentation domain the notion that bilingual exposure does not alter the pattern of acquisition as observed in monolingual populations (Werker and Byers-Heinlein, 2008).

To sum up, results from this research (a) offer evidence of an early ability for monosyllabic word segmentation in syllable-timed languages such as Spanish and Catalan, not previously described in the literature; (b) reveal no differences between monolingual and bilingual participants in this task, probably because both languages in the bilingual environment share the same rhythmic properties; and (c) show a specific developmental pattern that is compatible with an interpretation based on the facilitation effect that can be observed when rhythmic properties of the language match with the units to be extracted from fluent speech. These results should be the basis for further research exploring disyllabic word segmentation in the same linguistic population. They can also offer relevant information for future cross-linguistic research and they should be useful in studies comparing normally developing infants and clinical groups at risk for language delays in speech segmentation tasks.

### Conflict of interest statement

The authors declare that the research was conducted in the absence of any commercial or financial relationships that could be construed as a potential conflict of interest.

## Footnotes

^1^In a different study focused on the segmentation of monosyllabic verb forms by Canadian-French learning infants, positive evidence of segmentation, also based on a familiarity preference, was obtained at 11 months of age, but not earlier (Marquis and Shi, 2008).

## References

[B1] Abercrombie D. (1967). Elements of General Phonetics. Edinburgh: University of Edinburgh Press

[B2] Águila E. Ramon M. Pons F. Bosch L. (2005). Efecto de la exposición bilingüe sobre el desarrollo léxico inicial [Effect of bilingual exposure on early lexical development], in Estudios Sobre la Adquisición del Lenguaje, eds Mayor Cinca M. A. Zubiauz de Pedro B. Díez-Villoria E. (Salamanca, Spain: Ediciones Universidad de Salamanca), 676–692

[B3] Bortfeld H. Morgan J. L. Golinkoff R. M. Rathbun K. (2005). Mommy and me: familiar names help launch babies into speech stream segmentation. Psychol. Sci. 16, 298–304 10.1111/j.0956-7976.2005.01531.x 15828977 PMC2981583

[B4] Bosch L. Sebastián-Gallés N. (1997). Native-language recognition abilities in 4-month-old infants from monolingual and bilingual environments. Cognition 65, 33–69 10.1016/S0010-0277(97)00040-1 9455170

[B5] Bosch L. Sebastián-Gallés N. (2001). Evidence of early language discrimination abilities in infants from bilingual environments. Infancy 2, 29–49 10.1207/S15327078IN0201_3 33451225

[B6] Bosch L. Sebastián-Gallés N. (2003). Simultaneous bilingualism and the perception of a language-specific vowel contrast in the first year of life. Lang. Speech 46, 217–243 14748445 10.1177/00238309030460020801

[B7] Cutler A. Carter D. (1987). The predominance of strong initial syllables in the English vocabulary. Comput. Speech Lang. 2, 133–142

[B8] Fernald A. Kuhl P. K. (1987). Acoustic determinants of infant preference for motherese speech. Infant. Behav. Dev. 10, 279–293

[B9] Gómez R. (2002). Variability and detection of invariant structure. Psychol. Sci. 13, 431–436 12219809 10.1111/1467-9280.00476

[B10] Gout A. (2001). Etapes Précoces de l'acquisition du Lexique. Unpublished dissertation. Ecole des Hautes Etudes en Sciences Sociales, Paris, France

[B11] Goyet L. de Schonen S. Nazzi T. (2010). Words and syllables in fluent speech segmentation by French-learning infants: an ERP study. Brain Res. 1332, 75–89 10.1016/j.brainres.2010.03.047 20331982

[B12] Hirsh-Pasek K. Kemler Nelson D. G. Jusczyk P. W. Wright Cassidy K. Druss B. Kennedy L. (1987). Clauses are perceptual units for young infants. Cognition 26, 269–286 3677573 10.1016/s0010-0277(87)80002-1

[B13] Höhle B. Weissenborn J. (2003). German-learning infants' ability to detect unstressed closed class elements in continuous speech. Dev. Sci. 6, 122–127

[B14] Houston D. M. Jusczyk P. W. Kuijpers C. Coolen R. Cutler A. (2000). Cross-language word segmentation by 9-month-olds. Psychon. Bull. Rev. 7, 504–509 11082857 10.3758/bf03214363

[B15] Houston D. M. Santelmann L. M. Jusczyk P. W. (2004). English-learning infants' segmentation of trisyllabic words from fluent speech. Lang. Cogn. Proc. 19, 97–136

[B16] Hunter M. A. Ames E. W. (1988). A multifactor model of infant preferences for novel and familiar stimuli. Adv. Infancy Res. 5, 69–95

[B18] Johnson E. Seidl A. (2008). At eleven months, prosody still outranks statistics. Dev. Sci. 11, 1–11 19120421 10.1111/j.1467-7687.2008.00740.x

[B17] Johnson E. K. Jusczyk P. W. (2001). Word segmentation by 8-month-olds: when speech cues count more than statistics. J. Mem. Lang. 44, 548–567

[B19] Junge C. Kooijman V. Hagoort P. Cutler A. (2012). Rapid recognition at 10 months as a predictor of language development. Dev. Sci. 15, 463–473 10.1111/j.1467-7687.2012.1144.x 22709396

[B20] Jusczyk P. W. (1999). How infants begin to extract words from speech. Trends Cogn. Sci. 3, 323–328 10.1016/S1364-6613(99)01363-7 10461194

[B21] Jusczyk P. W. Aslin R. (1995). Infant's detection of the sound patterns words in fluent speech. Cogn. Psychol. 29, 1–23 10.1006/cogp.1995.1010 7641524

[B22] Jusczyk P. W. Houston D. M. Newsome M. (1999a). The beginnings of word segmentation in English-learning infants. Cogn. Psychol. 39, 159–207 10.1006/cogp.1999.0716 10631011

[B23] Jusczyk P. W. Hohne E. A. Bauman A. (1999b). Infants' sensitivity to allophonic cues for word segmentation. Percept. Psychophys. 61, 1465–1476 10598463 10.3758/bf03213111

[B24] Kuijpers C. Coolen R. Houston D. Cutler A. (1998). Using the head-turning technique to explore cross-linguistic performance differences, in Advances in Infancy Research, Vol. 12, eds Rovee-Collier C. Lippsitt L. Hyane H. (London: Ablex), 205–220

[B25] Ladefoged P. (1975). A Course in Phonetics. New York, NY: Harcourt Brace Jovanovich

[B26] Low E. L. Grabe E. Nolan F. (2000). Quantitative characterisations of speech rhythm: syllable-timing in Singapore English. Lang. Speech 43, 377–401 11419223 10.1177/00238309000430040301

[B27] Marquis A. Shi R. (2008). Segmentation of verb forms in preverbal infants. J. Acoust. Soc. Am. 123, EL105–EL110 10.1121/1.2884082 18396918

[B28] Mattys S. L. Jusczyk P. W. (2001a). Do infants segment words or recurring contiguous patterns? J. Exp. Psychol. Hum. Percept. Perform. 27, 644–655 11424651 10.1037//0096-1523.27.3.644

[B29] Mattys S. L. Jusczyk P. W. (2001b). Phonotactic cues for segmentation of fluent speech by infants. Cognition 78, 91–121 10.1016/S0010-0277(00)00109-8 11074247

[B30] Mattys S. L. Jusczyk P. W. Luce P. A. Morgan J. L. (1999). Phonotactic and prosodic effects on word segmentation in infants. Cogn. Psychol. 38, 465–494 10.1006/cogp.1999.0721 10334878

[B31] Nazzi T. Iakimova G. Bertoncini J. Frédonie S. Alcantara C. (2006). Early segmentation of fluent speech by infants acquiring French: emerging evidence for crosslinguistic differences. J. Mem. Lang. 54, 283–299

[B32] Nazzi T. Jusczyk P. W. Johnson E. K. (2000a). Language discrimination by English learning 5-month olds: effects of rhythm and familiarity. J. Mem. Lang. 43, 1–19

[B33] Nazzi T. Kemler Nelson D. Jusczyk P. Jusczyk A. M. (2000b). Six-month-olds' detection of clauses embedded in continuous speech: effects of prosodic well-formedness. Infancy 1, 123–147 10.1207/S15327078IN0101_11 32680315

[B34] Nespor M. (1990). On the rhythm parameter in phonology, in Logical Issues in Language Acquisition, ed Roca I. M. (Dordrecht: Foris), 157–175

[B35] Nespor M. Shukla M. Mehler J. (2011). Stress-timed vs. syllable-timed languages, in The Blackwell Companion to Phonology, Vol. II, eds Van Oostendorp M. Ewen C. J. Hume E. V. Rice K. (Chichester, UK: Blackwell Publication Inc.), 1147–1157

[B36] Newman R. S. Ratner N. B. Jusckzyk A. M. Jusckzyk P. W. Dow K. A. (2006). Infant's early ability to segment the conversational speech signal predicts later language development: a retrospective analysis. Dev. Psychol. 42, 643–655 10.1037/0012-1649.42.4.643 16802897

[B37] Payne E. Post B. Astruc L. Prieto P. Vanrell M. (2009). Rhythmic modification in child directed speech. Oxf. Univ. Work. Pap. Ling. Philol. Phon. 12, 123–144

[B38] Pelucchi B. Hay J. F. Saffran J. R. (2009). Statistical learning in a natural language by 8-month-old infants. Child Dev. 80, 674–685 10.1111/j.1467-8624.2009.01290.x 19489896 PMC3883431

[B40] Polka L. Sundara M. (2003). Word segmentation in monolingual and bilingual infant learners of English and French, in Proceedings of the 15th International Congress of Phonetic Sciences, eds Sole M. J. Recasens D. Romero J. (Barcelona: Causal Productions), 1021–1024

[B39] Polka L. Sundara M. (2012). Word segmentation in monolingual infants acquiring Canadian English and Canadian French: native language, cross-dialect, and cross-language comparisons. Infancy 17, 198–232 10.1111/j.1532-7078.2011.00075.x 32693528

[B41] Prieto P. Vanrell M. Astruc L. Payne E. Post B. (2012). Phonotactic and phrasal properties of speech rhythm. Evidence from Catalan, English, and Spanish. Speech Commun. 54, 681–702

[B42] Ramus F. Nespor M. Mehler J. (1999). Correlates of linguistic rhythm in the speech signal. Cognition 73, 265–292 10.1016/S0010-0277(99)00058-X 10585517

[B43] Rost G. C. McMurray B. (2009). Speaker variability augments phonological processing in early word learning. Dev. Sci. 12, 339–349 10.1111/j.1467-7687.2008.00786.x 19143806 PMC3011987

[B44] Sebastián-Gallés N. Bosch L. (2009). Developmental shift in the discrimination of vowel contrasts in bilingual infants: is the distributional account all there is to it? Dev. Sci. 12, 874–887 10.1111/j.1467-7687.2009.00829.x 19840043

[B45] Seidl A. (2007). Infants' use and weighting of prosodic cues in clause segmentation. J. Mem. Lang. 57, 24–48 10.1111/j.1467-7687.2008.00704.x 18576967

[B46] Seidl A. Cristià A. (2008). Developmental changes in the weighting of prosodic cues. Dev. Sci. 11, 596–606 10.1111/j.1467-7687.2008.00704.x 18576967

[B47] Seidl A. Johnson E. K. (2006). Infant word segmentation revisited: edge alignment facilitates target extraction. Dev. Sci. 9, 565–573 10.1111/j.1467-7687.2006.00534.x 17059453

[B48] Singh L. Reznick J. S. Xuehua L. (2012). Infant word segmentation and childhood vocabulary development: a longitudinal analysis. Dev. Sci. 15, 482–495 10.1111/j.1467-7687.2012.01141.x 22709398 PMC3383643

[B49] Soderstrom M. Seidl A. Kemler Nelson D. G. Jusczyk P. W. (2003). The prosodic bootstrapping of phrases: evidence from prelinguistic infants. J. Mem. Lang. 49, 249–267

[B50] Thiessen E. D. Hill E. A. Saffran J. R. (2005). Infant-directed speech facilitates word segmentation. Infancy 7, 53–71 10.1207/s15327078in0701_5 33430544

[B51] Thiessen E. D. Saffran J. R. (2003). When cues collide: use of stress and statistical cues to word boundaries in 7- to 9-month-old infants. Dev. Psychol. 39, 706–716 12859124 10.1037/0012-1649.39.4.706

[B52] Tranel B. (1987). The Sounds of French: An Introduction. Cambridge: Cambridge University Press

[B53] Werker J. F. Byers-Heinlein K. (2008). Bilingualism in infancy: first steps in perception and comprehension. Trends Cogn. Sci. 12, 144–151 10.1016/j.tics.2008.01.008 18343711

[B54] White L. Mattys S. L. (2007). Calibrating rhythm: first language and second language studies. J. Phon. 35, 501–522

[B55] White L. Mattys S. L. Wiget L. (2012). Language categorization by adults is based on sensitivity to durational cues, not rhythm class. J. Mem. Lang. 66, 665–679

